# Association of osteopontin with specific prognostic factors and survival in adjuvant breast cancer trials of the Hellenic Cooperative Oncology Group

**DOI:** 10.1186/s12967-017-1134-7

**Published:** 2017-02-13

**Authors:** Amanda Psyrri, Konstantine T. Kalogeras, Ralph M. Wirtz, George Kouvatseas, Georgia Karayannopoulou, Anna Goussia, Flora Zagouri, Elke Veltrup, Eleni Timotheadou, Helen Gogas, Angelos Koutras, Georgios Lazaridis, Christos Christodoulou, George Pentheroudakis, Panagiota Economopoulou, Apostolos Laskarakis, Petroula Arapantoni-Dadioti, Anna Batistatou, Maria Sotiropoulou, Gerasimos Aravantinos, Pavlos Papakostas, Paris Kosmidis, Dimitrios Pectasides, George Fountzilas

**Affiliations:** 1 Division of Oncology, Second Department of Internal Medicine, Attikon University Hospital, Athens, Greece; 2 Laboratory of Molecular Oncology, Hellenic Foundation for Cancer Research, Aristotle University of Thessaloniki, Thessaloníki, Greece; 3 Translational Research Section, Hellenic Cooperative Oncology Group, Data Office, Athens, Greece; 4 STRATIFYER Molecular Pathology GmbH, Cologne, Germany; 5 Department of Biostatistics, Health Data Specialists Ltd, Athens, Greece; 6 Department of Pathology, Faculty of Medicine, School of Health Sciences, Aristotle University of Thessaloniki, Thessaloníki, Greece; 7 Department of Pathology, Ioannina University Hospital, Ioannina, Greece; 8 Department of Clinical Therapeutics, Alexandra Hospital, School of Medicine, National and Kapodistrian University of Athens, Athens, Greece; 9 Department of Medical Oncology, Faculty of Medicine, Papageorgiou Hospital, School of Health Sciences, Aristotle University of Thessaloniki, Thessaloníki, Greece; 10 First Department of Medicine, Laiko General Hospital, School of Medicine, National and Kapodistrian University of Athens, Athens, Greece; 11 Division of Oncology, Department of Medicine, University Hospital, University of Patras Medical School, Patras, Greece; 12 Second Department of Medical Oncology, Metropolitan Hospital, Piraeus, Greece; 13 Department of Medical Oncology, Ioannina University Hospital, Ioannina, Greece; 14 First Department of Medical Oncology, Metropolitan Hospital, Piraeus, Greece; 15 Department of Pathology, Micromedica Labs, Athens, Greece; 16 Department of Pathology, Alexandra Hospital, Athens, Greece; 17 Second Department of Medical Oncology, Agii Anargiri Cancer Hospital, Athens, Greece; 18 Oncology Unit, Hippokration Hospital, Athens, Greece; 19 Second Department of Medical Oncology, Hygeia Hospital, Athens, Greece; 20 Oncology Section, Second Department of Internal Medicine, Hippokration Hospital, Athens, Greece; 21 Aristotle University of Thessaloniki, Thessaloníki, Greece

**Keywords:** Osteopontin, mRNA, Breast cancer, qRT-PCR, IHC, Prognostic value, Survival

## Abstract

**Background:**

The shift towards an earlier diagnosis of breast cancer (BC) highlights the need for biomarkers that would identify patients at risk for relapse and metastatic spread and indicate the potential value of additional treatment strategies. Osteopontin (OPN) is a matricellular protein that has been suggested to be a potential biomarker in BC. In the present study, we used archived BC patient samples to assess the clinical utility of OPN.

**Methods:**

Formalin-fixed paraffin-embedded tumor tissue samples from 975 patients were collected from two large phase III randomized adjuvant chemotherapy trials (HE10/97 and HE10/00) that included patients with high risk BC. All tissue samples were assessed for ER, PgR, Ki67 and HER2 protein expression. OPN protein and mRNA expression was evaluated using immunohistochemistry and quantitative reverse transcription-polymerase chain reaction, respectively.

**Results:**

OPN mRNA expression data were available for 814 patients, whereas OPN protein expression data were available for 546 patients. The majority of patients were ER/PgR-positive (78.3%), HER2-negative (76.5%) and Ki67-positive (55.2%) and had received adjuvant radiation therapy (76.8%) and hormonal therapy (81.1%). OPN mRNA expression was significantly associated with age (60.9% in high OPN tumors vs. 54.1% in low OPN tumors, p = 0.047), ER/PgR-negative status (25.7 vs. 17.2%, p = 0.004) and BC subtypes (p = 0.021). In addition, high OPN mRNA expression was significantly associated with reduced DFS (HR 1.26, 95% CI 1.00–1.59, Wald’s p = 0.050) and OS (HR 1.37, 95% CI 1.05–1.78, p = 0.019), while it retained its prognostic significance for both DFS (HR 1.39, 95% CI 1.10–1.77, p = 0.007) and OS (HR 1.54, 95% CI 1.61–2.05, p = 0.003) in the multivariate analysis.

**Conclusions:**

We showed that high OPN mRNA expression is associated with decreased DFS and OS in a large cohort of BC patients treated with adjuvant chemotherapy in a clinical trial setting. Our results suggest that OPN may serve as a prognostic factor and a potential target for therapy.

*Trial registration* Australian New Zealand Clinical Trials Registry; HE10/97 ACTRN12611000506998; HE10/00 ACTRN12609001036202

**Electronic supplementary material:**

The online version of this article (doi:10.1186/s12967-017-1134-7) contains supplementary material, which is available to authorized users.

## Background

The process of metastasis is responsible for the death of most patients with primary breast cancer. In clinical practice, to identify those patients at high risk of dying from metastatic spread, a series of prognostic pathologic factors are used. In addition, evaluation of biomarkers that may potentially play a role in tumor pathology allows physicians to prospectively select breast cancer patients for specific therapies. Currently, we use three predictive companion diagnostic tests for treatment selection: estrogen receptors (ER) and progesterone receptors (PgR) for endocrine therapy and human epidermal growth factor receptor 2 (HER2/neu) for anti-HER2 therapies. Despite the use of these three biomarkers for personalized risk stratification, a substantial proportion of patients still die of the disease. Therefore, we must identify additional biomarkers in order to improve the prognosis of breast cancer patients.

In addition to genetically altered cancer cells, a tumor mass consists of various normal cells that actively contribute to tumor progression, including the metastatic process. Tumor and stromal cells generate the extracellular matrix (ECM), which comprises structural proteins and proteins that regulate cell function and their interactions, such as osteopontin (OPN, *SPP1*) and other matricellular proteins. Matricellular proteins are involved in several cellular processes including cell adhesion and migration, ECM deposition, cell proliferation and survival [1]. All these processes are also important for primary tumor growth and metastasis, in which matricellular proteins are frequently aberrantly expressed. In addition, in a recent study using *SPP1* ^−/−^ mice along with gene silencing in tumor cells, OPN produced by tumor cells supported their survival in the blood stream, whereas both tumor- and host-derived OPN, particularly from myeloid cells, rendered the metastatic site more immunosuppressive [2].

Although OPN is not tumor-specific, its potential as a tumor biomarker has been demonstrated in several malignancies, including breast cancer [3, 4]. Elevated OPN expression has been associated with poor survival of cancer patients with different tumor histotypes [5–8]. Moreover, high OPN plasma concentrations were found in patients with metastatic breast cancer compared to healthy volunteers [9], while a number of studies have demonstrated that OPN may be associated with breast cancer progression and metastasis [10–12]. However, emerging data regarding the ability of OPN to predict overall survival (OS) and disease-free survival (DFS) in breast cancer patients have been inconsistent [13–15]. In the present study, we seek to determine the clinical utility of osteopontin in early breast cancer. To accomplish our goal we used archived samples from two large phase III randomized adjuvant trials in breast cancer patients.

## Methods

### Patient population

This was a retrospective translational research study among 1681 high-risk early breast cancer patients, enrolled in two prospective phase III trials. The HE10/97 trial [16] was a randomized phase III trial (ACTRN12611000506998) in patients with intermediate/high-risk operable breast cancer, comparing four cycles of epirubicin (E) followed by four cycles of intensified CMF (E-CMF) with three cycles of E, followed by three cycles of paclitaxel (T, Taxol^®^, Bristol Myers-Squibb, Princeton, NJ) followed by three cycles of intensified CMF (E-T-CMF). The current definition of high-risk breast cancer is based on the “International expert consensus on the primary therapy of early breast cancer 2007” [17]. Specifically, high-risk patients were node-positive patients with 1–3 involved lymph nodes and ER and PgR absent, or HER2/neu gene overexpressed or amplified; or node-positive patients with 4 or more involved lymph nodes. The cycles were given every two weeks with G-CSF support. Dose intensity of all drugs in both treatment arms was identical, but cumulative doses and duration of chemotherapy period differed. In total, 595 eligible patients entered the study in a period of 3.5 years (1997–2000).

The HE10/00 trial [18, 19] was a randomized phase III trial (ACTRN12609001036202), in which patients were treated with E-T-CMF (exactly as in the HE10/97 trial) or with four cycles of epirubicin/paclitaxel (ET) combination (given on the same day) every three weeks followed by three cycles of intensified CMF every two weeks (ET-CMF). By study design, the cumulative doses and the chemotherapy duration were identical in the two arms but dose intensity of epirubicin and paclitaxel was double in the E-T-CMF arm. A total of 1086 eligible patients with node-positive operable breast cancer were accrued in a period of 5 years (2000–2005).

HER2-positive patients received trastuzumab upon relapse, as previously described [20]. Treatment schedules for the two studies are shown in Additional file 1: Table S1. Baseline characteristics and clinical outcomes of both trials have already been described [16, 18, 19, 21]. Primary tumor diameter, axillary nodal status and tumor grade were obtained from the pathology report. Clinical protocols were approved by local regulatory authorities, while the present translational research study was approved by the “Papageorgiou” Hospital Institutional Review Board (July 15, 2013) and the Bioethics Committee of the Aristotle University of Thessaloniki School of Medicine (December 18, 2013). All patients signed a study-specific written informed consent before randomization, which in addition to giving consent for the trial allowed the use of biological material for future research purposes. All clinical investigations related to the present study have been conducted according to the principles expressed in the Declaration of Helsinki.

### Tissue microarray (TMA) construction

**Fig. 1:**
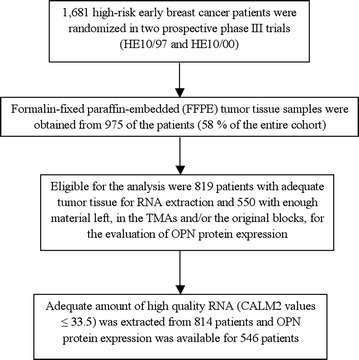
Remark diagram

Formalin-fixed paraffin-embedded (FFPE) tumor tissue samples from 975 patients (58.0% of 1681 randomized patients) were collected from both trials, retrospectively in the first (HE10/97) and prospectively in the second (HE10/00). The REMARK diagram [22] for the study is shown in Fig. 1. Hematoxylin-eosin stained sections from the tissue blocks were reviewed by two experienced breast cancer pathologists and the most representative tumor areas were marked for the construction of the ΤΜΑ blocks with the use of a manual arrayer (Model I, Beecher Instruments, San Prairie, WI), as previously described [23, 24]. Each case was represented by 2 tissue cores, 1.5 mm in diameter, obtained from the most representative areas of primary invasive tumors or in some cases (9.6%) from synchronous axillary lymph node metastases and re-embedded in 51 microarray blocks. Each TMA block contained 38–66 tissue cores from the original tumor tissue blocks, while cores from various neoplastic, non-neoplastic and reactive tissues were also included, serving as orientation controls for slide-based assays. Cases not represented, damaged or inadequate on the TMA sections were re-cut from the original blocks, when material was available, and these sections were used for protein expression analysis.

### Immunohistochemistry (IHC)

Immunohistochemical labeling was performed according to standard protocols on serial 2.5 μm thick sections from the original blocks or the TMA blocks. To assure optimal reactivity, immunostaining was applied 7–10 days after sectioning at the Laboratory of Molecular Oncology of the Hellenic Foundation for Cancer Research, Aristotle University of Thessaloniki School of Medicine. The staining procedures for HER2 (A0485 polyclonal antibody, Dako, Glostrup, Denmark), estrogen receptor (ER, clone 6F11, Novocastra™, Leica Biosystems, Newcastle, U.K), progesterone receptor (PgR, clone 1A6, Novocastra™, Leica Biosystems) and Ki67 (clone MIB-1, Dako) were performed using a Bond Max™ autostainer (Leica Microsystems, Wetzlar, Germany), as previously described in detail [25–28]. The staining procedures for OPN (clone OP3 N, code NCL-O-PONTIN, Novocastra™, Leica Biosystems) were also performed using a Bond Max™ autostainer, as previously described [29].

### Interpretation of the IHC results

The evaluation of all IHC sections was done by two experienced breast cancer pathologists, blinded as to the patients’ clinical characteristics and survival data, according to existing established criteria, as previously described [20]. Briefly, HER2 protein expression was scored in a scale from 0 to 3+, the latter corresponding to uniform, intense membrane staining in >30% invasive tumor cells [30]; ER and PgR were evaluated using the Histoscore method (max score: 400) and were considered positive if staining was present in ≥1% of tumor cell nuclei [31]; for Ki67, the expression was defined as low (<20%) or high (≥20%) based on the percentage of stained/unstained nuclei from the tumor areas [32]; and, OPN protein expression was scored in a scale from 0 to 8, using the Allred scoring system, with scores 5–8 being considered to be high expression [14, 33]. If one of the tissue cores was lost or damaged the overall score was determined from the remaining one. When whole tissue sections were used, the entire tumor area was evaluated. Of the 975 FFPE tumor tissue samples collected, only 550 (56.4%) had enough material left, in the TMAs and/or the original blocks, for the evaluation of OPN protein expression needed for the present study.

### Fluorescence in situ hybridization (FISH)

TMA sections or whole tissue sections (5 μm thick) were used for FISH analysis, using the ZytoLight^®^ SPEC *HER2*/*TOP2A*/CEP17 triple color probe (ZytoVision, Bremerhaven, Germany), as previously described [34]. FISH was performed according to the manufacturer’s protocol with minor modifications in all cases, not only the HER2 IHC 2+ cases. Four carcinoma cell lines (MDA-MB-231, MDA-MB-175, MDA-MB-453 and SK-BR-3) from the Oracle HER2 Control Slide (Leica Biosystems), with a known *HER2* gene status, were also used as a control for the FISH assays and analyzed for *HER2* genomic status.

For all probes, sequential (5 planes at 1.0 μm) digital images were captured using the Plan Apo VC 100×/1.40 oil objective (Nikon, Japan) using specific filters for each probe. The resulting images were reconstructed using specifically developed software for cytogenetics (XCyto-Gen, ALPHELYS, Plaisir, France). Processed sections were considered eligible for FISH evaluation according to the ASCO/CAP criteria [30]. For the evaluation of the *HER2* gene status, non-overlapping nuclei from the invasive part of the tumor were randomly selected, according to morphological criteria using DAPI staining, and scored. The virtual slides of HER2, ER or PgR stains, created as previously described [25], were used for selecting the invasive part of the tumor in each TMA. Twenty tumor nuclei were counted according to Press et al. [35]. The *HER2* gene was considered to be amplified when the *HER2*/CEP17 ratio was >2.2 [30], or the mean *HER2* copy number was >6 [36]. In cases with values at or near the cut-off (1.8–2.2), 20–40 additional nuclei were counted and the ratio was recalculated. In cases with a borderline ratio, additional FISH assays were performed in whole sections [37]. The data from the evaluation of *TOP2A* gene status were neither analyzed nor presented in the present manuscript. All primary image data of the TMA and whole tumor sections have been digitally scanned and made publicly available at: https://figshare.com/articles/Photos_of_TMA_and_whole_tumor_sections/3485879

### RNA isolation and quantitative reverse transcription-polymerase chain reaction (qRT-PCR) assessment

Prior to RNA isolation, macrodissection of tumor areas was performed in most (69%) of the FFPE sections (all sections with <50% tumor cell content). More than one FFPE section (2–8 sections, 10 μm thick) was used for RNA extraction when the tumor surface of a given sample was less than 0.25 cm^2^. From each FFPE section or macrodissected tissue fragments, RNA was extracted using a standardized fully automated isolation method for total RNA from FFPE tissue, based on germanium-coated magnetic beads (XTRAKT kit, STRATIFYER Molecular Pathology GmbH, Cologne, Germany) in combination with a liquid handling robot (XTRAKT XL, STRATIFYER Molecular Pathology GmbH), as previously described in detail [26, 28, 38, 39]. The method involves extraction-integrated deparaffinization and DNase I digestion steps. The quality and quantity of RNA was checked by measuring CALM2 expression as a surrogate for amplifiable mRNA by qRT-PCR. CALM2 was used as endogenous reference, since it had previously been identified as being highly and stably expressed among breast cancer tissue samples. Of the 975 FFPE tumor tissue samples collected, 819 (84.0%) had enough material left for RNA isolation needed for the present study.

qRT-PCR primers and labeled hydrolysis probes were selected using Primer Express^®^ Software, Version 2.2 and 3 (Applied Biosystems/Life Technologies, Karlsruhe, Germany), according to the manufacturer’s instructions, and were controlled for single nucleotide polymorphisms. All primers, probes and amplicons were checked for their specificity against nucleotide databases at NCBI using Basic Local Alignment Search Tool (BLAST). Primers and probes were purchased from Eurogentec S.A. (Seraing, Belgium). For each primer/probe set, the amplification efficiency was tested, aiming to reach comparable efficiency of >90% (efficiency range from 97.7 to 99.7%). Primers and hydrolysis probes were diluted to 100 µM, using a stock solution with nuclease-free water (Life Technologies GmbH, Darmstadt, Germany) [28, 39]. qRT-PCR was applied for the relative quantification (RQ) of OPN. The Primer/Probe (YakimaYellow/FAM-labeled) sets used for amplification of the target and reference genes were the following (5′ → 3′):

OPN probe CGACCAAGGAAAACT

- Forward primer CAGCCTTCTCAGCCAAACG
- Reverse primer CAAATCACTGCAATTCTCATGGTAGT

CALM2 probe TCGCGTCTCGGAAACCGGTAGC

- Forward primer GAGCGAGCTGAGTGGTTGTG
- Reverse primer AGTCAGTTGGTCAGCCATGCT

For PCR, 0.5 µM of each primer and 0.25 µM of each probe were used. All quantitative reverse-transcription PCRs were performed in triplicates using the SuperScript^®^ III Platinum^®^ One-Step qRT-PCR kit (Invitrogen/Life Technologies, Darmstadt, Germany) according to the manufacturer’s instructions. Experiments were performed on a Stratagene Mx3005p (Agilent Technologies, Waldbronn, Germany) with 30 min at 50 °C and 2 min at 95 °C followed by 40 cycles of 15 s at 95 °C and 30 s at 60 °C. The lengths of the amplicons detected by the OPN and CALM2 assays were 64 and 72 bp, respectively, with PCR efficiencies [E = 1^(10-slope)^] of 97.7 and 99.7%, respectively. Samples were considered eligible for further investigation (N = 814, Fig. 1) when the cycle threshold (CT) values of the housekeeping gene were ≤33.5 (triplicate mean values). Relative expression levels (relative quantification, RQ) of the target transcripts were calculated as 40-DCT values (DCT = mean CT target gene − mean CT housekeeping gene) to yield positively correlated numbers and to facilitate comparisons [28, 39]. A commercially available human reference RNA (Stratagene qPCR Human Reference Total RNA, Agilent Technologies, Waldbronn, Germany) was used as positive control. No-template controls were assessed in parallel to exclude contamination.

### Statistical analysis

DFS was defined as time from study entry to first tumor recurrence, secondary neoplasm, or death from any cause [40]. OS was measured from study entry until death from any cause. Surviving patients were censored at the date of last contact.

The prognostic value of OPN mRNA expression was examined in terms of DFS and OS, using the 50th percentile (median value) as the optimal cut-off and if this is not significant the upper and lower quartiles of the mRNA distribution were to be examined, as possible thresholds. In case a cut-off was of prognostic significance, it was used to dichotomize tumor expression into low and high. OPN protein expression was dichotomized into high versus low using the Allred 8-unit IHC scoring system (0–4: low vs. 5–8: high) [14]. The Fisher’s exact test or Pearson Chi square were used for group comparison of categorical data, while for continuous data the Mann–Whitney test was used. Kaplan–Meier curves and log-rank tests were used for comparing time to event distributions.

Univariate Cox proportional hazard regression analyses were performed for OPN mRNA and protein expression. Predictive significance of OPN mRNA and protein expression was examined by interaction tests between OPN mRNA or protein expression and chemotherapy treatment with paclitaxel (yes vs. no), hormonal therapy (yes vs. no) and radiation therapy (yes vs. no) using Cox regression models. In multivariate analysis, a backward selection procedure with a removal criterion p > 0.15 based on the likelihood ratio test was performed to identify significant clinicopathological variables among the following: age (≥50 vs. <50), nodal status (≥4 vs. 1–3 positive lymph nodes), tumor size (2–5 vs. ≤2 cm and >5 vs. ≤2 cm), radiation therapy (yes vs. no), hormonal therapy (yes vs. no), ER/PgR status (positive vs. negative), type of operation (modified radical mastectomy vs. breast-conserving surgery), chemotherapy treatment (E-T-CMF vs. E-CMF, ET-CMF vs. E-CMF) and *OPN* mRNA (high vs. low) or protein expression (high vs. low).

Results of this study were presented according to reporting recommendations for tumor marker prognostic studies [22]. This study is prospective-retrospective as described in Simon et al. [41]. The SAS software was used for statistical analysis (SAS for Windows, version 9.3, SAS Institute Inc., Cary, NC).

## Results

### Patient characteristics

OPN mRNA expression data were available for 814 patients, whereas OPN protein expression data were available for 546 patients. Basic clinical and pathological characteristics of patients are presented in Table 1. The majority of patients (centrally assessed by IHC and FISH in the case of HER2) were ER/PgR-positive (78.3%), HER2-negative (76.5%) and Ki67-positive (55.2%) and had received adjuvant radiation therapy (76.8%) and hormonal therapy (81.1%). High OPN mRNA expression was noted in 50% of the patients, while 89.2% of the patients had high OPN protein expression (Allred score 5–8).

**Table 1:** Selected patient and tumor characteristics

Characteristics	Total (N = 819)
	N (%)
Age (in years)	
<50	348 (42.5)
≥50	471 (57.5)
NPI score	
Median (range)	5.52 (3.0–9.0)
Treatment group	
E-CMF	123 (15.0)
E-T-CMF	392 (47.9)
ET-CMF	304 (37.1)
Menopausal status	
Premenopausal	375 (45.8)
Postmenopausal	444 (54.2)
Breast surgery	
Modified radical mastectomy	579 (70.7)
Breast-conserving surgery	240 (29.3)
ER/PgR status	
Negative	165 (21.7)
Positive	596 (78.3)
Histological grade	
I–II	406 (49.6)
III–Undifferentiated	413 (50.4)
Tumor size (cm)	
≤2	181 (22.8)
2–5	517 (65.2)
>5	95 (12.0)
Positive lymph nodes	
1–3 nodes	331 (40.5)
≥4 nodes	487 (59.5)
Adjuvant radiation therapy	
No	184 (23.2)
Yes	609 (76.8)
Adjuvant hormonal therapy	
No	154 (18.9)
Yes	661 (81.1)
Bone metastases	
No	711 (88.4)
Yes	93 (11.6)
HER2 status	
Negative	593 (76.5)
Positive	182 (23.5)
Ki67 protein expression	
Low (<20%)	367 (44.8)
High (≥20%)	452 (55.2)
Subtypes	
Luminal A	245 (32.3)
Luminal B	248 (32.8)
Luminal-HER2	99 (13.1)
HER2-enriched	79 (10.4)
Triple-negative	86 (11.4)
OPN mRNA expression	
Low (<50th percentile)	407 (50.0)
High (≥50th percentile)	407 (50.0)
OPN protein expression	
Low (0–4 Allred score)	59 (10.8)
High (5–8 Allred score)	487 (89.2)

### Normalized OPN mRNA expression

**Fig. 2:**
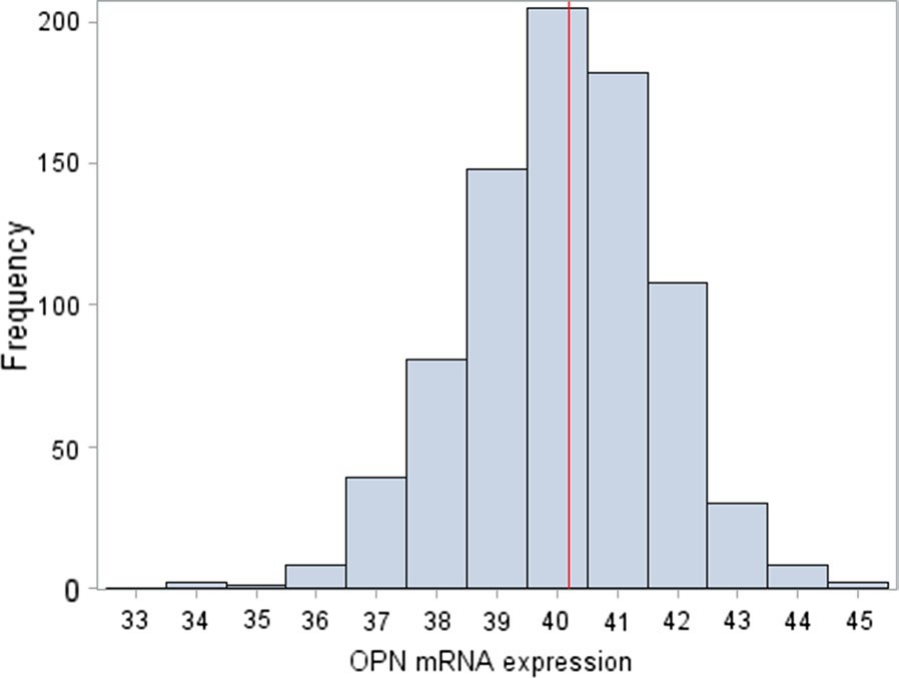
Distribution of OPN mRNA expression values. Median 40.2, range 33.5**–**44.7. *Red line* represents the 50th percentile (median)

The distribution of tumor samples according to the normalized expression of mRNA encoding for OPN is shown in Fig. 2. The median value for OPN mRNA expression was 40.2, with a range of 33.5–44.7. The 50th percentile (median value) was found to be the optimal cut-off for OPN mRNA expression in terms of both DFS and OS.

### Association of OPN mRNA expression with clinicopathologic parameters

OPN mRNA expression was significantly associated with age, as patients older than 50 years demonstrated higher OPN mRNA expression compared to patients younger than 50 years (Chi square, p = 0.047). In addition, postmenopausal and ER/PgR-negative patients were more frequent in the high OPN mRNA expressing group than in the low-expressing group (57.7 vs. 50.6%, Chi square, p = 0.041 and 25.7 vs. 17.2%, p = 0.004, respectively). Finally, there was a significant association between OPN mRNA expression and breast cancer subtypes (Chi square, p = 0.021), especially in triple-negative patients (14.5% in the high OPN mRNA expressing group vs. 8.0% in the low expressing group). There were no other significant associations between OPN mRNA expression and selected clinicopathological parameters (Table 2).

**Table 2:** Association of OPN mRNA expression with selected patient and tumor characteristics

	Low (N = 407)	High (N = 407)	*p* value
	N (%)	N (%)	
Age (in years)			
<50	187 (45.9)	159 (39.1)	*0.047*
≥50	220 (54.1)	248 (60.9)	
NPI score			
Median (range)	5.6 (3.0–7.8)	5.5 (3.2–9.0)	0.64
Treatment group			
E-CMF	69 (17.0)	53 (13.0)	0.18
E-T-CMF	196 (48.1)	192 (47.2)	
ET-CMF	142 (34.9)	162 (39.8)	
Menopausal status			
Premenopausal	201 (49.4)	172 (42.3)	*0.041*
Postmenopausal	206 (50.6)	235 (57.7)	
Type of operation			
Modified radical mastectomy	285 (70.0)	290 (71.3)	0.70
Breast conserving surgery	122 (30.0)	117 (28.7)	
ER/PgR status			
Negative	65 (17.2)	98 (25.7)	*0.004*
Positive	312 (82.8)	283 (74.3)	
Histological grade			
I–II	211 (51.8)	194 (47.7)	0.23
III	196 (48.2)	213 (52.3)	
Tumor size (cm)			
≤2	122 (30.0)	126 (31.0)	0.85
2–5	235 (57.7)	236 (58.0)	
>5	50 (12.3)	45 (11.0)	
Positive lymph nodes			
1–3 nodes	154 (37.9)	175 (43.0)	0.14
≥4 nodes	252 (62.1)	232 (57.0)	
Adjuvant RT			
No	80 (20.6)	103 (25.8)	0.081
Yes	309 (79.4)	296 (74.2)	
Adjuvant HT			
No	70 (17.3)	84 (20.7)	0.21
Yes	335 (82.7)	321 (79.3)	
Bone metastases			
No	363 (90.5)	346 (86.7)	
Yes	38 (9.5)	53 (13.3)	0.090
HER2 status^a^			
Negative	290 (75.5)	300 (77.5)	0.51
Positive	94 (24.5)	87 (22.5)	
Ki67 protein expression			
Low (<20%)	186 (45.7)	179 (44.0)	
High (≥20%)	221 (54.3)	228 (56.0)	0.62
Subtypes			
Luminal A	125 (33.3)	120 (31.8)	*0.021*
Luminal B	128 (34.0)	119 (31.5)	
Luminal-HER2	58 (15.4)	41 (10.8)	
HER2-enriched	35 (9.3)	43 (11.4)	
Triple-negative	30 (8.0)	55 (14.5)	

Comparisons were made using Chi square or Fisher’s exact tests

*RT* radiation therapy, *HT* hormonal therapy

Significant p values are shown in italics

^a^Positive HER2 status: HER2 3+ by IHC or HER2 amplification by FISH

### Prognostic/predictive value of OPN mRNA and OPN protein expression

Survival status of all patients was updated in June 2014. The median follow-up time was 119.9 months (range 0.1–191.9). During this time, 291 patients (35.5%) had developed documented disease progression and 226 (27.6%) had died.

**Fig. 3:**
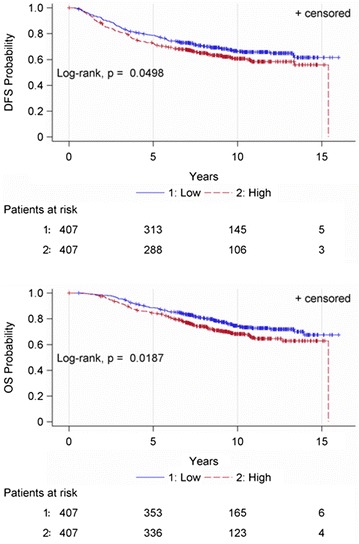
Disease-free survival (DFS) and overall survival (OS) according to OPN mRNA expression

Univariate Cox regression analysis showed that high OPN mRNA expression (above the 50th percentile) was associated with increased risk for relapse (HR 1.26, 95% CI 1.00–1.59, Wald’s p = 0.050) and death (HR 1.37, 95% CI 1.05–1.78, p = 0.019). Kaplan–Meier curves for DFS and OS according to OPN mRNA expression are shown in Fig. 3.

OPN mRNA expression was not predictive for benefit from the addition of paclitaxel to the E-CMF regimen either for DFS (interaction p = 0.97) or OS (interaction p = 0.88). In addition, OPN mRNA expression was not predictive for benefit either from hormonal therapy (DFS interaction p = 0.76 and OS p = 0.57) or from radiation therapy (DFS interaction p = 0.73 and OS p = 0.67).

With regard to prognostic value, OPN protein expression was not significantly associated with either DFS (HR 0.81, 95% CI 0.52–1.26, p = 0.35) or OS (HR 0.78, 95% CI 0.47–1.27, p = 0.32). The predictive value of OPN protein expression for benefit from the addition of paclitaxel to the E-CMF regimen was not examined due to the fact that one subgroup (E-CMF/low OPN protein expression) contained only one patient. OPN protein expression was not predictive for benefit either from hormonal therapy (DFS interaction p = 0.27 and OS p = 0.95) or from radiation therapy (DFS interaction p = 0.97 and OS p = 0.97).

### Multivariate analysis

In the multivariate Cox regression analysis, shown in Table 3, OPN mRNA expression retained its prognostic significance for both DFS (HR 1.34, 95% CI 1.04–1.71, Wald’s p = 0.022) and OS (HR 1.54, 95% CI 1.16–2.05, p = 0.003). Four or more positive nodes and greater tumor size were found to be associated with higher risk for relapse and death, while adjuvant hormonal therapy was a favorable prognostic factor for both DFS and OS.

**Table 3:** Multivariate Cox regression analysis

Disease-free survival	HR	95% CI	p value
Number of positive nodes			
≥4 versus 1–3	2.51	1.88–3.63	<0.001
Tumor size			
2–5 cm versus ≤2 cm	1.45	1.08–1.95	0.013
>5 cm versus ≤2 cm	1.65	1.10–2.48	0.015
Adjuvant HT			
Yes versus no	0.69	0.51–0.93	0.016
OPN mRNA expression			
High versus low	1.34	1.04–1.71	0.022
*Overall survival*			
Number of positive nodes			
≥4 versus 1–3	3.59	2.48–5.19	<0.001
Tumor size			
2–5 cm versus ≤2 cm	1.59	1.13–2.34	0.009
>5 cm versus ≤2 cm	1.87	1.18–2.97	0.008
Adjuvant HT			
Yes versus no	0.55	0.39–0.76	<0.001
OPN mRNA expression			
High versus low	1.54	1.16–2.05	0.003

*HR* hazard ratio, *CI* confidence interval

## Discussion

In the present study, we sought to determine the prognostic significance of OPN mRNA and protein expression in archived samples from two large phase III randomized adjuvant trials in breast cancer patients. At the time our adjuvant studies were started, OPN as a potential tumor biomarker in breast cancer was categorized as “+” on the Tumor Marker Utility Grading System (TMUGS) published by Hayes et al. [4], defined as “sufficient data are available to demonstrate that the marker correlates with the biologic process and/or biologic endpoint related to the use and that the marker results might affect favorable clinical outcome for that use”. However, the marker is still considered investigational and should not be used for standard clinical practice. We demonstrated that OPN mRNA expression inversely correlated with DFS and OS, while it retained its independent prognostic significance in multivariate analysis for increased risk for both relapse and death. OPN protein expression assessed by conventional IHC did not show an association with relapse and death. Regarding predictive significance, OPN mRNA and protein expression were not found to be predictive for benefit from paclitaxel chemotherapy, hormonal therapy or radiation therapy in our patients.

In our study, we found that OPN mRNA expression was inversely associated with DFS and OS. Our study is the first, to our knowledge, to examine the prognostic significance of OPN mRNA and protein expression in breast cancer samples derived from patients treated in a clinical trial setting. A handful of studies have assessed the prognostic value of OPN in early breast cancer and our results are in agreement with these studies. Ortiz-Martínez et al. [42] analyzed by RT-PCR 309 breast cancer samples and 6 breast cancer cell lines for OPN mRNA expression, its splicing variant-c and OPN protein expression. The median fold change of total OPN mRNA expression was higher in HER2-positive and triple-negative/basal-like tumors, whereas OPN-c mRNA expression was upregulated in the triple-negative/basal-like subtype. We also found a significant association between OPN mRNA expression and breast cancer subtypes (p = 0.021), especially in triple-negative patients. In the study by Ortiz-Martinez et al. [42] DFS was significantly shorter for patients whose tumors overexpressed total OPN (67 vs. 73%) but in multivariate analysis only OPN-c mRNA expression emerged as a significant predictor for relapse. Moreover, increased *OPN*-*c* stratified subgroups of patients at higher risk of recurrence among immunophenotypes, especially in the triple-negative/basal-like subtype (risk of relapse 70% in patients with low OPN-expression vs. 83% in patients with OPN-c overexpression). Patani et al. [43] analyzed 127 breast carcinomas and 33 normal tissues for *OPN* transcript levels using real-time PCR. OPN-a mRNA expression decreased with increasing TNM stage and was associated worse clinical outcome. OPN-b mRNA expression increased with histological grade and Nottingham Prognostic Index (NPI) stage, was higher in patients who died of breast cancer than in those who were disease-free after 10 years and predicted DFS. OPN-c mRNA expression was associated with histological grade and poor prognosis. Furthermore, high expression levels predicted local recurrence, worse DFS and bone metastases [43].

Although OPN mRNA expression was associated with survival, OPN protein expression was not. The discordant findings can be explained by several factors. Firstly, all mRNAs are not equally translated into proteins [44]. Secondly, microRNAs repress numerous genes by inhibiting mRNA translation into protein, while several post-translational mechanisms control protein turnover and abundance [44]. Finally, it appears that there is a stronger evolutionary pressure for constant protein levels compared to constant mRNA levels (reviewed in [44]). The above factors would also explain the lack of correlation between OPN mRNA expression and OPN protein expression observed in our study.

Many studies have assessed the prognostic value of OPN protein expression in breast cancer and have yielded conflicting results [33, 45–50]. A recent meta-analysis demonstrated that high OPN protein expression was positively associated with lymph node metastases (pooled odds ratio 2.03, 95% CI 1.20–3.43, p = 0.008) and decreased OS (HR 3.69, 95% CI 1.45–9.42, p < 0.001, random-effects model) and DFS (pooled HR 2.40, 95% CI 1.27–4.55, p = 0.007, fixed-effects model) [51]. We did not however find a significant association of OPN protein status with either DFS or OS.

Our findings suggest a possible relationship between the ER signaling pathway and OPN mRNA expression. Coexpression of OPN and ERa has previously been reported in breast cancer patients [45, 48]. The association between OPN and two other estrogen inducible proteins, pS2 and PgR, was also significant in the above study by Rudland et al. [48]. These associations suggest molecular connections between OPN and the ER signaling pathway [52].

Our study has several strengths. There were strong laboratory and clinical data supporting our interest in exploring OPN as a biomarker in breast cancer. The patient population was well defined, since all patients included in the study were accrued in the context of two randomized adjuvant trials. All important patient and tumor characteristics were presented and were also included in the multivariate analyses. Both randomized trials had a long follow-up. Finally, to our knowledge, this is the largest study evaluating the prognostic impact of OPN mRNA and protein expression in early breast cancer.

A major limitation of our study is that this is a retrospective study; our results should therefore be confirmed in validation cohorts, applying the OPN mRNA cut-off used the present study. In addition, protein expression of OPN splice variants should also be evaluated by immunohistochemistry, as suggested in a recent study by Zduniak et al. [3].

## Conclusions

In conclusion, we demonstrated that high OPN mRNA expression is associated with reduced DFS and OS in a large cohort of breast cancer patients treated with adjuvant chemotherapy in a randomized clinical trial setting. This finding has implications for the prognostic classification of patients, the use of dose-dense chemotherapy regimens in all patients with high OPN mRNA expression, as well as the use of novel more efficacious treatments when they become available in the future. In addition, our results suggest that OPN may serve as a potential molecular target for the treatment of breast cancer patients.

## Additional file

**Additional file 1.** OPN supplementary data.

## Abbreviations

CMF: cyclophosphamide methotrexate fluorouracil
CT: cycle threshold
DFS: disease-free survival
E: epirubicin
ECM: extracellular matrix
ER: estrogen receptors
ET: epirubicin–paclitaxel
FFPE: formalin-fixed paraffin-embedded
FISH: fluorescence in situ hybridization
G-CSF: granulocyte-colony stimulating factors
HER: human epidermal growth factor receptor
IHC: immunohistochemistry
mRNA: messenger RNA
OPN: osteopontin
OS: overall Survival
PgR: progesterone receptors
qRT-PCR: quantitative reverse transcription-polymerase chain reaction
RNA: ribonucleic acid
RQ: relative quantification
SPP1: osteopontin gene
T: paclitaxel
TMA: tissue microarray

## References

[1] Wong GS, Rustgi AK (2013). Matricellular proteins: priming the tumour microenvironment for cancer development and metastasis. Br J Cancer.

[2] Sangaletti S, Tripodo C, Sandri S, Torselli I, Vitali C, Ratti C, Botti L, Burocchi A, Porcasi R, Tomirotti A (2014). Osteopontin shapes immunosuppression in the metastatic niche. Cancer Res.

[3] Zduniak K, Agrawal A, Agrawal S, Hossain MM, Ziolkowski P, Weber GF (2016). Osteopontin splice variants are differential predictors of breast cancer treatment responses. BMC Cancer.

[4] Hayes DF, Bast RC, Desch CE, Fritsche H, Kemeny NE, Jessup JM, Locker GY, Macdonald JS, Mennel RG, Norton L (1996). Tumor marker utility grading system: a framework to evaluate clinical utility of tumor markers. J Natl Cancer Inst.

[5] Shi SM, Su ZB, Zhao JJ, Yu DJ, Tu JW, Zhu JQ, Zhao JP, Sheng L, Wang SB, Sheng YJ (2016). Increased osteopontin protein expression may be correlated with poor prognosis in non-small-cell lung cancer: a meta analysis. J Cancer Res Ther.

[6] Luo SD, Chen YJ, Liu CT, Rau KM, Chen YC, Tsai HT, Chen CH, Chiu TJ (2015). Osteopontin involves cisplatin resistance and poor prognosis in oral squamous cell carcinoma. Biomed Res Int.

[7] Zhao M, Liang F, Zhang B, Yan W, Zhang J (2015). The impact of osteopontin on prognosis and clinicopathology of colorectal cancer patients: a systematic meta-analysis. Sci Rep.

[8] Kiss T, Ecsedi S, Vizkeleti L, Koroknai V, Emri G, Kovacs N, Adany R, Balazs M (2015). The role of osteopontin expression in melanoma progression. Tumour Biol.

[9] Singhal H, Bautista DS, Tonkin KS, O’Malley FP, Tuck AB, Chambers AF, Harris JF (1997). Elevated plasma osteopontin in metastatic breast cancer associated with increased tumor burden and decreased survival. Clin Cancer Res.

[10] Furger KA, Menon RK, Tuck AB, Bramwell VH, Chambers AF (2001). The functional and clinical roles of osteopontin in cancer and metastasis. Curr Mol Med.

[11] Xu K, Tian X, Oh SY, Movassaghi M, Naber SP, Kuperwasser C, Buchsbaum RJ (2016). The fibroblast Tiam1-osteopontin pathway modulates breast cancer invasion and metastasis. Breast Cancer Res.

[12] Sharon Y, Raz Y, Cohen N, Ben-Shmuel A, Schwartz H, Geiger T, Erez N (2015). Tumor-derived osteopontin reprograms normal mammary fibroblasts to promote inflammation and tumor growth in breast cancer. Cancer Res.

[13] Bramwell VH, Tuck AB, Chapman JA, Anborgh PH, Postenka CO, Al-Katib W, Shepherd LE, Han L, Wilson CF, Pritchard KI (2014). Assessment of osteopontin in early breast cancer: correlative study in a randomised clinical trial. Breast Cancer Res.

[14] Thorat D, Sahu A, Behera R, Lohite K, Deshmukh S, Mane A, Karnik S, Doke S, Kundu GC (2013). Association of osteopontin and cyclooxygenase-2 expression with breast cancer subtypes and their use as potential biomarkers. Oncol Lett.

[15] Patani N, Jiang W, Mokbel K (2008). Osteopontin C mRNA expression is associated with a poor clinical outcome in human breast cancer. Int J Cancer.

[16] Fountzilas G, Skarlos D, Dafni U, Gogas H, Briasoulis E, Pectasides D, Papadimitriou C, Markopoulos C, Polychronis A, Kalofonos HP (2005). Postoperative dose-dense sequential chemotherapy with epirubicin, followed by CMF with or without paclitaxel, in patients with high-risk operable breast cancer: a randomized phase III study conducted by the Hellenic Cooperative Oncology Group. Ann Oncol.

[17] Goldhirsch A, Wood WC, Gelber RD, Coates AS, Thurlimann B, Senn HJ (2007). Progress and promise: highlights of the international expert consensus on the primary therapy of early breast cancer 2007. Ann Oncol.

[18] Fountzilas G, Dafni U, Gogas H, Linardou H, Kalofonos HP, Briasoulis E, Pectasides D, Samantas E, Bafaloukos D, Stathopoulos GP (2008). Postoperative dose-dense sequential chemotherapy with epirubicin, paclitaxel and CMF in patients with high-risk breast cancer: safety analysis of the Hellenic Cooperative Oncology Group randomized phase III trial HE 10/00. Ann Oncol.

[19] Gogas H, Dafni U, Karina M, Papadimitriou C, Batistatou A, Bobos M, Kalofonos HP, Eleftheraki AG, Timotheadou E, Bafaloukos D (2012). Postoperative dose-dense sequential versus concomitant administration of epirubicin and paclitaxel in patients with node-positive breast cancer: 5-year results of the Hellenic Cooperative Oncology Group HE 10/00 phase III Trial. Breast Cancer Res Treat.

[20] Razis E, Bobos M, Kotoula V, Eleftheraki AG, Kalofonos HP, Pavlakis K, Papakostas P, Aravantinos G, Rigakos G, Efstratiou I (2011). Evaluation of the association of PIK3CA mutations and PTEN loss with efficacy of trastuzumab therapy in metastatic breast cancer. Breast Cancer Res Treat.

[21] Fountzilas G, Dafni U, Bobos M, Batistatou A, Kotoula V, Trihia H, Malamou-Mitsi V, Miliaras S, Chrisafi S, Papadopoulos S (2012). Differential response of immunohistochemically defined breast cancer subtypes to anthracycline-based adjuvant chemotherapy with or without paclitaxel. PLoS ONE.

[22] McShane LM, Altman DG, Sauerbrei W, Taube SE, Gion M, Clark GM (2006). Statistics subcommittee of NCIEWGoCD: reporting recommendations for tumor MARKer prognostic studies (REMARK). Breast Cancer Res Treat.

[23] Kononen J, Bubendorf L, Kallioniemi A, Barlund M, Schraml P, Leighton S, Torhorst J, Mihatsch MJ, Sauter G, Kallioniemi OP (1998). Tissue microarrays for high-throughput molecular profiling of tumor specimens. Nat Med.

[24] Skacel M, Skilton B, Pettay JD, Tubbs RR (2002). Tissue microarrays: a powerful tool for high-throughput analysis of clinical specimens: a review of the method with validation data. Appl Immunohistochem Mol Morphol.

[25] Fountzilas G, Kourea HP, Bobos M, Televantou D, Kotoula V, Papadimitriou C, Papazisis KT, Timotheadou E, Efstratiou I, Koutras A (2011). Paclitaxel and bevacizumab as first line combined treatment in patients with metastatic breast cancer: the Hellenic Cooperative Oncology Group experience with biological marker evaluation. Anticancer Res.

[26] Psyrri A, Kalogeras KT, Kronenwett R, Wirtz RM, Batistatou A, Bournakis E, Timotheadou E, Gogas H, Aravantinos G, Christodoulou C (2012). Prognostic significance of UBE2C mRNA expression in high-risk early breast cancer. A Hellenic Cooperative Oncology Group (HeCOG) Study. Ann Oncol.

[27] Fountzilas G, Dafni U, Bobos M, Kotoula V, Batistatou A, Xanthakis I, Papadimitriou C, Kostopoulos I, Koletsa T, Tsolaki E (2013). Evaluation of the prognostic role of centromere 17 gain and HER2/topoisomerase II alpha gene status and protein expression in patients with breast cancer treated with anthracycline-containing adjuvant chemotherapy: pooled analysis of two Hellenic Cooperative Oncology Group (HeCOG) phase III trials. BMC Cancer.

[28] Stavridi F, Kalogeras KT, Pliarchopoulou K, Wirtz RM, Alexopoulou Z, Zagouri F, Veltrup E, Timotheadou E, Gogas H, Koutras A (2016). Comparison of the ability of different clinical treatment scores to estimate prognosis in high-risk early breast cancer patients: a Hellenic Cooperative Oncology Group Study. PLoS ONE.

[29] Tuck AB, O’Malley FP, Singhal H, Harris JF, Tonkin KS, Kerkvliet N, Saad Z, Doig GS, Chambers AF (1998). Osteopontin expression in a group of lymph node negative breast cancer patients. Int J Cancer.

[30] Wolff AC, Hammond ME, Schwartz JN, Hagerty KL, Allred DC, Cote RJ, Dowsett M, Fitzgibbons PL, Hanna WM, Langer A (2007). American Society of Clinical Oncology/College of American Pathologists guideline recommendations for human epidermal growth factor receptor 2 testing in breast cancer. J Clin Oncol.

[31] Hammond ME, Hayes DF, Dowsett M, Allred DC, Hagerty KL, Badve S, Fitzgibbons PL, Francis G, Goldstein NS, Hayes M (2010). American Society of Clinical Oncology/College Of American Pathologists guideline recommendations for immunohistochemical testing of estrogen and progesterone receptors in breast cancer. J Clin Oncol.

[32] Cheang MC, Chia SK, Voduc D, Gao D, Leung S, Snider J, Watson M, Davies S, Bernard PS, Parker JS (2009). Ki67 index, HER2 status, and prognosis of patients with luminal B breast cancer. J Natl Cancer Inst.

[33] Pang H, Lu H, Song H, Meng Q, Zhao Y, Liu N, Lan F, Liu Y, Yan S, Dong X, Cai L (2013). Prognostic values of osteopontin-c, E-cadherin and beta-catenin in breast cancer. Cancer Epidemiol.

[34] Bartlett JM, Munro AF, Dunn JA, McConkey C, Jordan S, Twelves CJ, Cameron DA, Thomas J, Campbell FM, Rea DW (2010). Predictive markers of anthracycline benefit: a prospectively planned analysis of the UK National Epirubicin Adjuvant Trial (NEAT/BR9601). Lancet Oncol.

[35] Press MF, Sauter G, Buyse M, Bernstein L, Guzman R, Santiago A, Villalobos IE, Eiermann W, Pienkowski T, Martin M (2011). Alteration of topoisomerase II-alpha gene in human breast cancer: association with responsiveness to anthracycline-based chemotherapy. J Clin Oncol.

[36] Bempt IV, Van Loo P, Drijkoningen M, Neven P, Smeets A, Christiaens MR, Paridaens R, De Wolf-Peeters C (2008). Polysomy 17 in breast cancer: clinicopathologic significance and impact on HER-2 testing. J Clin Oncol.

[37] Sauter G, Lee J, Bartlett JM, Slamon DJ, Press MF (2009). Guidelines for human epidermal growth factor receptor 2 testing: biologic and methodologic considerations. J Clin Oncol.

[38] Pentheroudakis G, Kotoula V, Eleftheraki AG, Tsolaki E, Wirtz RM, Kalogeras KT, Batistatou A, Bobos M, Dimopoulos MA, Timotheadou E (2013). Prognostic significance of ESR1 gene amplification, mRNA/protein expression and functional profiles in high-risk early breast cancer: a translational study of the Hellenic Cooperative Oncology Group (HeCOG). PLoS ONE.

[39] Pentheroudakis G, Raptou G, Kotoula V, Wirtz RM, Vrettou E, Karavasilis V, Gourgioti G, Gakou C, Syrigos KN, Bournakis E (2015). Immune response gene expression in colorectal cancer carries distinct prognostic implications according to tissue, stage and site: a prospective retrospective translational study in the context of a hellenic cooperative oncology group randomised trial. PLoS ONE.

[40] Hudis CA, Barlow WE, Costantino JP, Gray RJ, Pritchard KI, Chapman JA, Sparano JA, Hunsberger S, Enos RA, Gelber RD, Zujewski JA (2007). Proposal for standardized definitions for efficacy end points in adjuvant breast cancer trials: the STEEP system. J Clin Oncol.

[41] Simon RM, Paik S, Hayes DF (2009). Use of archived specimens in evaluation of prognostic and predictive biomarkers. J Natl Cancer Inst.

[42] Ortiz-Martinez F, Perez-Balaguer A, Ciprian D, Andres L, Ponce J, Adrover E, Sanchez-Paya J, Aranda FI, Lerma E, Peiro G (2014). Association of increased osteopontin and splice variant-c mRNA expression with HER2 and triple-negative/basal-like breast carcinomas subtypes and recurrence. Hum Pathol.

[43] Patani N, Jouhra F, Jiang W, Mokbel K (2008). Osteopontin expression profiles predict pathological and clinical outcome in breast cancer. Anticancer Res.

[44] Kendrick N (2014). A gene’s mRNA level does not usually predict its protein level.

[45] de Silva Rudland S, Martin L, Roshanlall C, Winstanley J, Leinster S, Platt-Higgins A, Carroll J, West C, Barraclough R, Rudland P (2006). Association of S100A4 and osteopontin with specific prognostic factors and survival of patients with minimally invasive breast cancer. Clin Cancer Res.

[46] Kim YW, Park YK, Lee J, Ko SW, Yang MH (1998). Expression of osteopontin and osteonectin in breast cancer. J Korean Med Sci.

[47] Ribeiro-Silva A, Oliveira da Costa JP (2008). Osteopontin expression according to molecular profile of invasive breast cancer: a clinicopathological and immunohistochemical study. Int J Biol Markers.

[48] Rudland PS, Platt-Higgins A, El-Tanani M, De Silva Rudland S, Barraclough R, Winstanley JH, Howitt R, West CR (2002). Prognostic significance of the metastasis-associated protein osteopontin in human breast cancer. Cancer Res.

[49] Wang X, Chao L, Ma G, Chen L, Tian B, Zang Y, Sun J (2008). Increased expression of osteopontin in patients with triple-negative breast cancer. Eur J Clin Invest.

[50] Tuck AB, Chambers AF (2001). The role of osteopontin in breast cancer: clinical and experimental studies. J Mammary Gland Biol Neoplasia.

[51] Xu YY, Zhang YY, Lu WF, Mi YJ, Chen YQ (2015). Prognostic value of osteopontin expression in breast cancer: a meta-analysis. Mol Clin Oncol.

[52] El-Tanani M, Fernig DG, Barraclough R, Green C, Rudland P (2001). Differential modulation of transcriptional activity of estrogen receptors by direct protein-protein interactions with the T cell factor family of transcription factors. J Biol Chem.

