# Supplementary material for: Association of osteopontin with specific prognostic factors and survival in adjuvant breast cancer trials of the Hellenic Cooperative Oncology Group
Source: J Transl Med. 2017 Feb 13;15:30. doi: 10.1186/s12967-017-1134-7 (PMC5304396; doi:10.1186/s12967-017-1134-7)
Supplement: Supplementary file 1 — Additional file 1. OPN supplementary data. [file 12967_2017_1134_MOESM1_ESM.doc]

**Supplemental Table S1: Clinical trial characteristics.**

| **Trial** | **Accrual period** | **N** | **n** | **Treatment schedule** | **Eligibility criteria** | **References** |
| --- | --- | --- | --- | --- | --- | --- |
| **HE10/97  Australian New Zealand Clinical Trials Registry ACTRN12611000506998** | **1997 - 2000** | **595** | **277** | **E-T-CMF:** Epirubicin 110 mg/m^2^ q 2 weeks x 3 followed by paclitaxel 250 mg/m^2^ q 2 weeks x 3 followed by cyclophosphamide 840 mg/m^2^, methotrexate 57 mg/m^2^, fluorouracil 840 mg/m^2^ (CMF) q 2 weeks x 3. GCSF support in all cycles.  **E-CMF:** Epirubicin 110 mg/m^2^ q 2 weeks x 4 followed by CMF q 2 weeks x 4. GCSF support in all cycles.  Patients with ER/PgR-positive tumors received tamoxifen 20 mg daily for five years. Premenopausal patients received additional treatment with an LH-RH analog for two years. All patients who underwent partial mastectomy or with tumors >5 cm and/or with ≥ 4 infiltrated axillary nodes, irrespectively of the type of surgery, were irradiated. Radiation therapy and hormonal therapy were administered after the completion of chemotherapy. | Eligible were women with: histologically confirmed epithelial breast cancer; pathological stage T1-3 N1 M0 or T3 N0 M0; Eastern Cooperative Oncology Group performance status 0-1; normal cardiac function; and adequate bone marrow, hepatic and renal function. | Fountzilas G, Skarlos D, et al. Postoperative dose-dense sequential chemotherapy with epirubicin, followed by CMF with or without paclitaxel, in patients with high-risk operable breast cancer: a randomized phase III study conducted by the Hellenic Cooperative Oncology Group.  Ann Oncol. 2005;16(11):1762-71. |
| **HE10/00  Australian New Zealand Clinical Trials Registry ACTRN12609001036202** | **2000 - 2005** | **1,086** | **698** | **E-T-CMF:** As in the HE10/97 trial.  **ET-CMF:** Epirubicin 83 mg/m^2^ + Paclitaxel 187 mg/m^2^ q 3 weeks x 4 followed by cyclophosphamide 840 mg/m^2^, methotrexate 57 mg/m^2^, fluorouracil 840 mg/m^2^ (CMF) q 2 weeks x 3. GCSF support in all cycles.  Premenopausal patients received hormonal therapy as in the HE10/97 trial. Postmenopausal patients received tamoxifen 20 mg daily for 2-3 years followed 2-3 years of daily examestane 25 mg. Criteria for irradiation were the same as in the HE10/97 trial. | Eligible were women with: histologically confirmed epithelial breast cancer; pathological stage T1-4 N1-2 M0; Eastern Cooperative Oncology Group performance status 0-1; normal cardiac function; and adequate bone marrow, hepatic and renal function. | Gogas H, Dafni U, et al. Postoperative dose-dense sequential versus concomitant administration of epirubicin and paclitaxel in patients with node-positive breast cancer: 5-year results of the Hellenic Cooperative Oncology Group HE 10/00 phase III Trial. Breast Cancer Res Treat. 2012;132(2):609-19. |

N, number of patients enrolled in the trials; n, number of patients included in the current study with tumor tissue blocks available.
